# Systemic and Ocular Predictive Factors in the Treatment of Diabetic Macular Edema with Bevacizumab

**DOI:** 10.3390/medicina62020283

**Published:** 2026-01-30

**Authors:** Esen Cakmak Cengiz, Ozlem Eski Yucel

**Affiliations:** 1 Clinic of Ophthalmology, Ünye State Hospital, Ordu 52300, Turkey; 2 Department of Ophthalmology, Faculty of Medicine, Ondokuz Mayıs University, Samsun 55270, Turkey; drozlem38@hotmail.com

**Keywords:** diabetic macular edema, bevacizumab, biomarker, optical coherence tomography

## Abstract

*Background and Objectives:* This study aimed to explore peripheral blood and OCT risk biomarkers that may modify the anatomical and functional response to intravitreal bevacizumab (IVB) treatment in diabetic macular edema (DME). *Materials and Methods:* The study included patients with non-proliferative diabetic retinopathy (NDR) who had not previously undergone laser photocoagulation or IVB. Data on demographics, hemogram, and biochemistry within one month before treatment were collected. Best corrected visual acuity (BCVA), intraocular pressure (IOP), and spectral-domain OCT (SD-OCT) measurements were recorded before and after three monthly IVB injections. OCT parameters included central macular thickness (CMT), inner and outer retinal thickness (IRT, ORT), ganglion cell layer thickness (GCT), and central choroidal thickness (CCT). *Results:* The study analyzed 48 eyes. Significant improvements were seen in BCVA (logMAR 0.44 to 0.18), while IOP increased slightly (15 to 17.5 mmHg). There were notable reductions in CMT, GCT, and IRT. Anatomical success (83.3%) was associated in univariate analysis with greater OCT improvement and higher white blood cell count (WBC) levels (*p* < 0.05). Central macular thickness decreased by 27% (from 427 to 312 μm), and visual acuity improved from 0.44 to 0.18 logMAR. In logistic regression analysis, factors associated with functional success (75%) included higher blood urea nitrogen (BUN) levels [OR 1.11 (95% CI: 1.03–1.21), *p* = 0.008], lower low-density lipoprotein (LDL) levels (*p* = 0.013), and lower baseline intraocular pressure (IOP) (*p* = 0.013). *Conclusions:* Intravitreal bevacizumab is effective in early diabetic macular edema. Elevated BUN and lower LDL levels may be associated with a favorable functional response to treatment

## 1. Introduction

Diabetic retinopathy (DR) is the most common ocular complication of diabetes mellitus (DM) and one of the essential causes of preventable blindness in individuals aged 20–74 years [1]. The most common cause of visual impairment in these patients is diabetic macular edema (DME). The incidence and severity of DR increases with increasing duration of diabetes [2]. DR is a multifactorial disease that includes hyperglycemia, chronic inflammation, increased vascular permeability, retinal ischemia, neovascularization, and neurodegeneration [3]. At any stage of DR, chronic inflammation leads to increased local and systemic levels of cell adhesion molecules, vascular endothelial growth factor (VEGF), and inflammatory molecules, which disrupt the blood–retinal barrier, leading to hemorrhage, exudation, and retinal edema [4].

Intravitreal anti-VEGF or corticosteroid injections, retinal grid, and focal laser photocoagulation applications are used to treat DME [5]. Glycosylated hemoglobin (HBA1c), hemogram, and biochemistry parameters analyzed in peripheral blood samples of DM patients differ from values observed in nondiabetic individuals/those of the healthy population [6,7,8]. Comorbid conditions such as chronic renal failure (CRF), hypertension (HT), hyperlipidemia (HL), and obesity are also frequently found in patients with DME. The presence of systemic diseases accompanying DM is an important factor that may affect the formation of DME and its response to treatment with anti-VEGF [9,10,11]. Recent studies have included investigation of biomarkers that can predict how patients with DME will respond to treatment. Optical Coherence Tomography (OCT) provides information about the qualitative and quantitative characteristics of the retinal layers. OCT features can be used in the diagnosis and follow-up of DME and also serve as biomarkers [12,13,14].

The vascular endothelial growth factor (VEGF) plays a central role in the pathogenesis of diabetic macular edema, and anti-VEGF agents currently form the basis of DME treatment. Among the most frequently used anti-VEGF drugs in clinical practice are ranibizumab, aflibercept, and, more recently, faricimab, which has a dual-target mechanism of action. These agents are effective in DME treatment due to their distinct binding profiles and durations of action. Bevacizumab, although off-label in many countries, remains widely used in clinical practice due to its cost-effectiveness and broad accessibility [15]. In this context, identifying biomarkers that predict anatomical and functional responses in patients treated with bevacizumab is clinically essential for treatment planning and anticipating potential early treatment changes.

This study aimed to determine demographic, biochemical, and OCT biomarkers affecting the anatomical and functional response to DME treatment with intravitreal bevacizumab (IVB).

## 2. Materials and Methods

### 2.1. Patient Population

The study followed the principles of the Declaration of Helsinki and was approved by the University Clinical Studies Ethics Committee. Number B.30.2.ODM.0.20.08/633.

The records of patients who received IVB (Avastin^®^; Genentech, Inc., South San Francisco, CA, USA) for the treatment of DME between May 2021 and September 2022 at the Ophthalmology Clinic of Ondokuz Mayıs University Medical Faculty Hospital were retrospectively reviewed. Patients with non-proliferative DR (NPDR) and DME who received three consecutive monthly doses of IVB; who had a best corrected visual acuity (BCVA) of ≥0.1 Snellen line and central macular thickness (CMT) of ≥300 μm before treatment; who had not received any intravitreal injection or retinal laser photocoagulation treatment before; who had at least one follow-up examination after IVB treatment; and who had hemogram, biochemistry, and glycosylated hemoglobin (HBA1c) evaluations at the time of initiation of IVB treatment were included in the study. In cases with bilateral DME, the eye with the higher CMT was included in the study. Patients with proliferative DR and/or ischemic maculopathy; those with retinal disease other than DR or other ocular diseases that could cause ME; who had received intravitreal injection, retinal laser photocoagulation, or vitreoretinal surgery before IVB treatment; who had not undergone hemogram, biochemistry, or HBA1c evaluations at the time of initiation of IVB treatment; and who were lost to follow-up after IVB treatment were excluded from the study.

### 2.2. Systemic Evaluation

Demographic data of the patients, such as age, gender, duration of DM (years), and accompanying systemic diseases (HT, HL, CRF) were recorded. Patients were classified into four groups according to the drugs they used for the treatment of DM before the first IVB injection: those who did not use systemic treatment; those who used only oral antidiabetic (OAD); those who used only insulin; and those who used OAD and insulin together. The patients’ body mass index (BMI) was calculated and recorded.

The results of peripheral blood samples of all patients taken up to 1 month before the first IVB injection were recorded: hemoglobin (Hb), hematocrit (Hct), white blood cell count (WBC), monocyte count, blood urea nitrogen (BUN), creatinine, triglyceride, low-density lipoprotein (LDL), high-density lipoprotein (HDL), fasting blood glucose (FBG), HBA1c, erythrocyte sedimentation rate (ESR), and C-Reactive Protein (CRP).

### 2.3. Assessment of Imaging

Pre-treatment fundus fluorescein angiography (FA) of all patients was reviewed by a retina specialist (Ö.E.Y.), and patients were selected according to inclusion and exclusion criteria after distinguishing between NPDR and proliferative DR (PDR). Multicolor fundus imaging, macular OCT, and foveal-centered Enhanced Depth Imaging (EDI) mode OCT imaging of all the patients were performed by the same person using the Heidelberg Spectralis OCT (Spectralis HRA+OCT: Heidelberg Engineering, Heidelberg, Germany) device at the pre-treatment and the first, second, and third month post-treatment visits. All images were evaluated and recorded by a retina specialist and an ophthalmologist (O.E.Y and E.Ç.C.).

CMT was obtained from the 30-degree macular OCT images with the G-FAST mode of Spectralis OCT, from the 1 × 1 mm central circle of the thickness map created with the automatic segmentation algorithm of Spectralis software (V 7.0.4). Inner retinal thickness (IRT), outer retinal thickness (ORT), and ganglion cell thickness (GCT) were recorded from the automatic segmentation analysis centered on the fovea, and the sum of these parameters was used to automatically calculate the CMT value on the same device. For central choroidal thickness (CCT), macular OCT obtained by a 30-degree radial scan protocol with the EDI mode of the Spectralis OCT was used. The value calculated by manually marking the distance between the outer border of the retinal pigment epithelium and the chorioscleral interface with the “draw region” tool of the device in the subfoveal area on the horizontal scan image was recorded as CCT. The type of edema and the presence of vitreomacular interface abnormalities were recorded from the OCT images. The edema was divided into diffuse or cystoid edema and was also recorded as subretinal fluid (SRF) and intraretinal fluid (IRF). Vitreomacular interface abnormalities were recorded as the presence of epiretinal membrane (ERM), vitreomacular traction (VMT), vitreomacular adhesion (VMA), disruption of the ellipsoid zone (EZ), retinal inner layer irregularity (DRIL), hard exudate (HE), and hyperreflective foci (HF).

### 2.4. Ophthalmological Examination

The best corrected visual acuity (BCVA) values were recorded from the ocular examinations of the patients at baseline and during the first, second, third month follow-up visits after IVB loading treatment. In addition, intraocular pressure (IOP) values, anterior segment examination findings, and dilated ophthalmoscopy examination findings were recorded. The patient complaints and side effects related to the treatment were also recorded.

Treatment responses were evaluated by comparing BCVA and CMT values of the patients 1 month after the third dose of IVB with the baseline values. The patients were grouped as anatomical and functional successful and unsuccessful: Anatomical success was defined as a ≥10% reduction in central macular thickness compared to baseline. Functional success was defined as a gain of ≥5 ETDRS letters after treatment. Patients were categorized into anatomically successful or unsuccessful and functionally successful or unsuccessful groups, which were then compared in terms of demographic data, peripheral blood parameters and OCT findings.

### 2.5. Statistical Analyses

Data were analyzed with IBM Statistical Package for the Social Sciences (SPSS) V23. The Shapiro–Wilk test was performed to verify the normality of the data. In independent paired groups, the independent two-sample t test was used to compare normally distributed data, and the Mann–Whitney U test was used to compare non-normally distributed data. To compare pre-treatment and post-treatment values, the paired two-sample *t* test was used for normally distributed data, and the Wilcoxon Test was used for non-normally distributed data. Yate’s correction, Fisher’s exact test, and Pearson’s chi-square test were used to compare categorical data. Binary Logistic Regression Analysis was used to determine risk factors affecting anatomical and functional failure. Analysis results were presented as frequency (%) for categorical variables, mean ± standard deviation, and median (minimum–maximum) for quantitative variables. The significance level was accepted as *p* < 0.050.

## 3. Results

### 3.1. Demographic and Systemic Data

In a total of 48 patients, 22 (45.8%) were female and 26 (54.2%) were male. Those who met the criteria were included in the study. The mean age of the patients was 64.02 ± 8.48 years, and the mean BMI was 29.38 ± 4.66 kg/m^2^. The mean duration of DM was 15.73 ± 7.86 years. All patients were receiving antidiabetic treatment, except three (6.3%) patients who were referred to endocrinology examination before starting IVB treatment and were diagnosed with DM. Twenty-two (45.8%) of the cases had HT and all of them were using antihypertensive drugs. The mean HBA1c was 9.1 ± 2.1 mmol/mol. Creatinine levels of seven (14.6%) patients were higher than the reference value, but none of the patients were undergoing hemodialysis. Forty-six (95.8%) patients had HL. Demographic and systemic characteristics of the study population are given in Table 1.

### 3.2. Ocular Examination and OCT Findings

Thirty-nine (81.2%) of the forty-eight eyes were phakic and nine (18.8%) were pseudophakic. The median BCVA, which was 0.44 (0.0–1.0) logMAR at baseline, decreased to 0.18 (0.0–1.0) logMAR after treatment (*p* < 0.001). The median IOP, which was 15 (10–23) mmHg at baseline, increased to 17.5 (10–23) mmHg after treatment (*p* = 0.040) (Table 2). The median CMT was 427 (280–699) micrometers (µm) at baseline and decreased to 312 (215–587) µm after treatment (*p* < 0.001). The median IRT (338.5 vs. 229.5 µm, *p* < 0.001) and GCT (31.5 vs. 25 µm, *p* < 0.001) were also significantly lower after treatment compared to at baseline. No statistically significant difference was detected between the baseline and post-treatment values of ORT and CCT. Baseline and post-treatment quantitative OCT parameters are given in Table 3, and the frequencies of baseline qualitative OCT parameters are given in Table 4.

### 3.3. Anatomical Success

**Figure 1:**
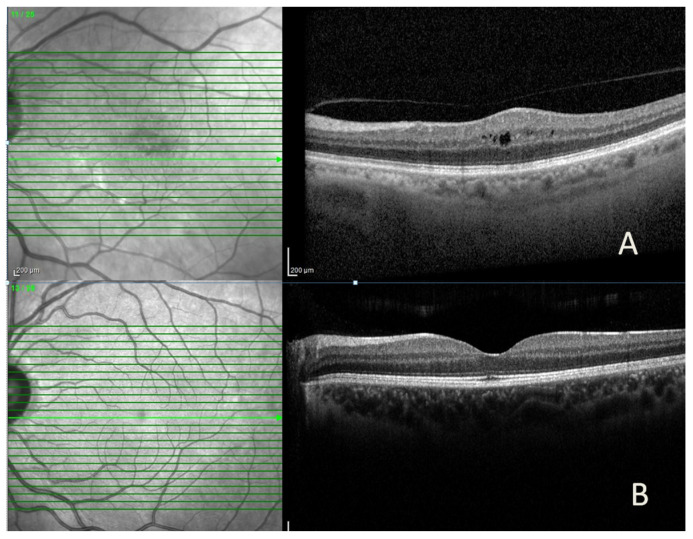
Representative spectral-domain optical coherence tomography (SD-OCT) images of a patient with diabetic macular edema, showing (**A**) intraretinal cyst and increased central macular thickness before intravitreal bevacizumab injection and (**B**) marked reduction in macular edema after treatment.

Anatomical success was achieved in 40 (83.3%) of the 48 cases, and complete resolution of ME (dry macula) was achieved in 18 (45%) (Figure 1). In eyes with anatomical success, the median CMT (314.5 vs. 459.6 µ, *p* = 0.008) and IRT (227.9 vs. 376 µ, *p* = 0.005) were statistically lower than those without. In contrast, the median GCT, ORT, and CCT were similar (Table 3). In eyes with anatomical success, the median CMT, IRT, GCT, and CCT decreased significantly after treatment compared to baseline, while median ORT did not change. In eyes without anatomical success, quantitative OCT parameters were similar before and after treatment (Table 3).

No significant difference was observed in demographic characteristics, systemic diseases (Table 1), and qualitative OCT parameters (Table 4) in cases with and without anatomical success (Table 1). Baseline median BCVA and IOP were similar in eyes with and without anatomical success. In eyes with anatomical success, the median BCVA (0.48 vs. 0.18 LogMAR, *p* < 0.001) and IOP (15 vs. 17.5 mmHg, *p* = 0.008) increased statistically compared to baseline. There was no similar change in eyes without anatomical success (Table 2). The median WBC (8.27 vs. 6.69 × 10^3^/µL, *p* = 0.049) was found to be higher in cases with anatomical success than in cases without anatomical success. There was no significant difference between the groups in terms of other peripheral blood parameters (Table 5).

### 3.4. Functional Success

Functional success was achieved in 36 (75%) of the 48 eyes. The median BCVA was similar in eyes with and without functional success. In eyes with functional success, the median BCVA increased statistically after IVB treatment compared to baseline (0.48 vs. 0.15 LogMAR, *p* < 0.001), while the increase in BCVA in eyes without functional success was not statistically significant (Table 2). In eyes with functional success, the median IOP was lower than in eyes without functional success (14.5 vs. 16 mmHg, *p* = 0.013) at baseline, but an increase was detected after IVB treatment compared to the baseline. (14.5 vs. 17 mmHg, *p* < 0.001) (Table 2). Demographic characteristics, systemic diseases (Table 1), baseline quantitative (Table 3), and qualitative (Table 4) OCT parameters were similar in cases with and without functional success. In eyes with functional success, median CMT (303 vs. 420.5 µ, *p* = 0.017), IRT (223.5 vs. 340 µ, *p* = 0.022), and ORT (83.6 vs. 89.8 µ, *p* = 0.008) were significantly lower after IVB treatment compared to eyes without functional success, while GCT and CCT were similar. In eyes with functional success, CMT (432 vs. 303 µm, *p* < 0.001), GCT (31.6 vs. 25 µm, *p* < 0.001), and IRT (344.7 vs. 223.5 µm, *p* < 0.001) decreased significantly after IVB treatment compared to baseline, while ORT and CCT did not change. In eyes without functional success, CMT (469 vs. 420.5 µm, *p* = 0.027) and IRT (384.9 vs. 340 µm, *p* = 0.015) decreased significantly after IVB treatment compared to baseline; ORT (82 vs. 89.8 µm, *p* = 0.023) significantly increased, but the decrease in GCT and CCT was not statistically significant (Table 3). In cases with the functional success, the mean BUN (33.9 vs. 22.6 mg/dL, *p* = 0.005) was found to be higher, while the mean LDL (106.3 vs. 153.83 mg/dL, *p* = 0.002) was found to be lower than in cases without functional success. There was no significant difference between the groups in terms of other peripheral blood parameters (Table 5).

### 3.5. Regression Analysis

In binary logistic regression analysis, demographic characteristics, systemic diseases, peripheral blood parameters, baseline BCVA, baseline IOP, and qualitative and quantitative OCT parameters were found to be not independent risk factors for anatomical success in the univariate model (Table 6). In the binary logistic regression analysis, demographic characteristics, systemic diseases, baseline BCVA, and qualitative and quantitative OCT parameters were not independent risk factors for functional success in the univariate model. It was found that a 1 mg/dL increase in BUN increased functional success by 1.11 times (*p* = 0.008), a 1 mg/dL increase in LDL was associated with a 1.203-fold decrease in functional success (*p* = 0.013), and a 1 mmHg increase in baseline IOP decreased functional success by 1.32 times (*p* = 0.013) (Table 7).

## 4. Discussion

Our study investigated the early results of IVB treatment in DME and systemic and ocular biomarkers that could allow us to predict the treatment outcome. The study revealed that anatomical success was achieved in 83.3% of cases and functional success was achieved in 75% of cases. Visual acuity was improved from 0.44 to 0.18 logMAR. Central macular thickness decreased by 27% from 427 to 312 µm. In the anatomically successful group, CMT decreased from 435 to 314 µm, and VA increased from 0.48 to 0.18 logMAR. In the functionally successful group, CMT decreased from 432 to 303 µm, and VA increased from 0.48 to 0.15 logMAR. It was determined that qualitative and quantitative OCT parameters did not affect anatomical and functional success. WBC levels were seen to be high in the anatomically successful group, but regression analysis could not confirm that WBC was a predictive factor for anatomical success. BUN levels were found to be higher and LDL levels were lower in the functionally successful group. Increases in LDL and IOP were found to be negative predictive factors, while an increase in BUN was a positive predictive factor for functional success.

Significant reductions were observed in ganglion cell thickness (GCT) and inner retinal thickness (IRT) following intravitreal bevacizumab (IVB) treatment, together with a decrease in central macular thickness (CMT). In contrast, outer retinal thickness (ORT) and central choroidal thickness (CCT) did not show significant changes. It has been reported that DME starts from the inner retinal layers and then progresses to the outer retinal layers [16,17]. Since our patients’ DR was not at an advanced stage and their DME was not chronic, it was concluded that ME affected IRT more, DRT was not affected yet, and therefore, thinning was achieved in the inner layers of the retina with the IVB treatment, except for total CMT. There was a significant difference in IRT between the groups with and without anatomical success but no difference in DRT after treatment. These findings confirm that the inner retinal layers contribute mainly to the increase in CMT in DME. Different experimental studies have reported that hypertrophy occurs in Müller cells even in the early stages of DR before the development of DME, and this situation is thought to play a key role in DR [18]. Muller cells cover almost the entire retina, and their cell nuclei are located in the inner nuclear layer. In the early stages of DME development, an increase in thickness occurs predominantly in the inner nuclear layer, probably due to the localization of the deep retinal vascular network in the same area and the accumulation of extracellular fluid in this area with the disruption of the blood–retinal barrier [16]. While a significant decrease in IRT was observed in patients with anatomical success, the lack of a significant decrease in ORT is probably due to the short duration of DME and early treatment in our patients.

In this study, it is seen that functional success was also achieved in patients with anatomical success. This situation suggests that the treatment response will be more effective in treatment-naïve patients, in cases where macular ischemia is not present, and when irreversible neuronal damage (neuronal apoptosis) that may occur in chronic DME has not developed. It has been reported that retinal ganglion cells in diabetic patients degenerate due to hyperglycemia and microvascular damage [19,20]. In a study in which DME was treated with the intravitreal conbercept, although the inner nuclear and outer nuclear layer thicknesses decreased the most, it was found that the decrease in GCT and inner plexiform layer thicknesses were the best indicators for BCVA [14].

A reduction in CCT was also detected in patients with anatomical success. It is thought that the inflammatory process in DR and DME causes an increase in choroidal thickness and that CCT decreases with treatment. CCT has been previously reported to decrease along with CMT in the treatment of DME with intravitreal anti-VEGF or steroids [21]. Our patient population in the study was the elderly and had chronic DM. In addition, their metabolic control was poor because BMI and HBA1c values were high. It can be seen that they are a patient group with a high inflammatory and oxidative stress load. Therefore, it was thought that a decrease was achieved with treatment in their probably thickened choroids.

In our study, predictive factors for anatomic treatment success were examined. Although WBC values were within the normal reference range in both patient groups, they were higher in patients with anatomic success than in those without anatomic success. Chatziralli et al. [22] showed that WBC level is not a prognostic factor for the response to anti-VEGF treatment in ME due to retinal vein occlusion, in which inflammatory cytokines play a role, such as DME. In chronic inflammatory diseases such as DM, WBC causes increased synthesis of inflammatory cytokines such as IL-6 and VEGF, which results in deterioration of the blood–retinal barrier and RPE function [23]. Considering that our patients with moderate WBC elevation showed good anatomical response to IVB treatment, it can be said that inflammatory cytokines secreted by WBC and its subtypes cause more vascular permeability and ME, thus responding better to treatment with anti-VEGF. In the recent study, it was determined that demographic characteristics and systemic diseases did not affect anatomical and functional success. Hwang et al. [9] divided the results of IVB treatment of DME into two groups as successful and unsuccessful based on the decrease in CMT and increase in BCVA. They reported that there was no significant difference between the groups in terms of age, gender, duration of DM, BMI, levels of triglyceride, LDL, HDL, and hemoglobin. Our findings also support these results. Functional success was achieved in 75% of the patients in this study. In the BEVORDEX study, the visual improvement rate of 10 or more letters in DME cases treated with IVB was found to be 40% [24]. However, since the gain of five or more letters was taken as the criterion for visual success in this study, our functional success rate was found to be higher. It has been reported that anatomical and functional success can be achieved in the early period of treatment with IVB, not only in treatment-naïve DME but also in persistent DME [25].

Anatomical success was also achieved in patients with functional success. In these cases, GCT and IRT decreased significantly, in addition to CMT. As previously discussed, the decrease in GCT may be related to neuronal recovery and contributes to visual gain. A reduction in ME was also observed in patients where functional success was not achieved. In these patients, while GCT and IRT decreased, a significant increase was observed in ORT. In order for light to be transmitted from the photoreceptors to the nerve fiber layer, each layer of the retina must be intact. Thickening of the inner retinal layers reduces the oxygen delivered to the outer retina and disrupts the metabolic activity of the outer retinal layers. In patients without functional success, an increase in ORT may indicate early photoreceptor damage, suggesting that visual acuity may remain unchanged or deteriorate further. The thicker (edematous) ORT affects the metabolism and oxygenation of the photoreceptor layer, and perhaps as the process becomes chronic, deterioration in the EZ may occur. In the treatment of DME, increasing ORT may be an indicator of treatment non-response. It may also be a criterion to consider alternative treatments such as different anti-VEGFs or steroids.

This study determined that BUN and LDL values could predict functional treatment success. BUN was significantly higher and LDL was significantly lower in patients with functional success than in patients without. However, other findings used in the staging of renal failure such as microalbumin, protein in urine, glomerular filtration rate were not evaluated in the present study. In addition, no significant difference was observed between patients with successful and unsuccessful treatment response in terms of creatinine level. When the literature was examined, it was reported that BUN, creatinine, and estimated glomerular filtration rate were negatively correlated with improvement in CMT in the treatment of DME with IVB or intravitreal dexamethasone [9]. However, it was also reported that kidney disease was not a predictive factor in the treatment of DME with ranibizumab but was associated with poor visual prognosis in the sham group [26]. In the RIDE and RISE studies, systemic factors affecting the visual recovery obtained with ranibizumab treatment of DME were examined, and it was determined that diabetes treatment, HbA1c, BMI, blood pressure, and renal function had no effects [10]. Regression analysis also confirmed that an increase in BUN alone, without renal failure, reduces the risk of functional failure. This result may be due to early recognition and treatment of DME in patients with increased BUN. So, these patients are referred to relevant specialists, including ophthalmologists, to be screened for microvascular complications after BUN elevation is detected in their routine endocrinological examinations.

There are studies in the literature examining the relationship between blood lipids and DR or DME. Some publications are showing that the severity of DME is not correlated with lipid levels, and there are also studies showing that the success of anti-VEGF treatment in DME is unrelated to LDL levels [26,27]. In this study, it was determined that LDL elevation is a negative prognostic factor for functional success. Since diabetic retinopathy develops due to microvascular damage, LDL may act as an occlusive agent in damaged areas of the endothelium and reduce retinal oxygenation. This situation may be related to cellular damage and decreased visual gain. In this study, a significant increase in IOP was observed after IVB treatment. Previous studies have reported no significant increase in IOP at 1 month after IVB or ranibizumab injection [28]. In the BEVORDEX study, IOP increases of up to 5 mmHg were observed in patients with DME treated with IVB during the 12-month follow-up period, but no IOP increase of 10 mmHg was detected in any patient [24]. Especially in elderly patients receiving IVB injections, IOP should be closely monitored and attention should be paid to IOP increases. The current literature reports that different anti-VEGF agents, such as ranibizumab, aflibercept, and faricimab, are effective in treating diabetic macular edema and provide anatomical and functional improvement [15]. However, prospective randomized studies are needed to compare different anti-VEGF agents directly. Our analysis focuses on biomarkers that can predict early treatment response in patients treated with bevacizumab, rather than comparative efficacy.

One limitation of the study is its retrospective design. The study was conducted with a relatively small number of cases because it included treatment-naive participants and had strict inclusion criteria. The relatively small number of patients in the anatomical (n = 8) and functional failure (n = 12) groups represents a limitation of the study. Larger cohorts and confirmation of these findings by independent research groups are warranted. Since staging was not performed for non-proliferative DR, its effect on treatment could not be evaluated. Although non-proliferative DR patients were included, the diameter and size of peripheral ischemia were not examined. Macular edema was detected at the patients’ first presentation, and the duration of DME could not be included in the analyses because there was no objective evidence of duration. Following DME diagnosis, some patients underwent endocrinology evaluations during treatment, and metabolic controls were also implemented; however, changes in peripheral blood parameters were not considered during follow-up. Another limitation of this study is that laboratory parameters were assessed only once at baseline. Serial measurements during follow-up could have provided additional insight into the dynamic relationship between systemic biomarkers and treatment response.

## 5. Conclusions

In this study, three consecutive monthly doses of IVB treatment provided 83.3% anatomical success, 75% functional success, and 45% complete resolution of ME (dry macula) in eyes with DME. Improvement was mainly due to thinning of the ganglion cell and inner retinal layers. In patients without improvement, there was an increase in ORT caused by the progression of edema from the inner retinal layers to the outer retinal layers. It was determined that LDL and IOP elevation were negative predictive factors for functional success, while only BUN elevation without renal failure was a positive predictive factor. Demographic data, systemic diseases, other peripheral blood parameters, pre-treatment VA, and qualitative and quantitative OCT parameters had no effect on treatment response. Identifying predictors of response before initiating DME treatment provides clinicians the opportunity to inform patients about anatomical and functional expectations and to develop personalized treatment strategies.

## Figures and Tables

**Table 1:** Demographic/systemic characteristics and their relationships with success.

			Anatomical Success			Functional Success		
			Success (n = 40)	Unsuccess (n = 8)	P^1^	Success (n = 36)	Unsucces (n = 12)	P^2^
Age (years)		64.02 ± 8.48/66 (43–78)	64.2 ± 8.85	63.13 ± 6.77	0.747 ^t^	63.9 ± 8.9	64.4 ± 7.2	0.990 ^U^
Gender	Female	22 (45.8)	17 (42.5)	5 (62.5)	0.442 ^F^	15 (41.7)	7 (58.3)	0.503 ^Y^
	Male	26 (54.2)	23 (57.5)	3 (37.5)		21 (58.3)	5 (41.7)	
BMI (kg/m^2^)		29.38 ± 4.66	28.8 ± 4.42	32.27 ± 5.04	0.053 ^t^	28.9 ± 4.5	30.9 ± 4.1	0.053 ^t^
DM duration (years)		15.73 ± 7.86	16 ± 8.08	14.38 ± 6.97	0.599 ^t^	16.6 ± 7.95	13.08 ± 7.25	0.240 ^U^
DM medication	No	3 (6.3)	2 (5)	1 (12.5)	0.748 ^P^	1 (2.8)	2 (16.7)	0.270 ^P^
	Insulin	3 (6.3)	3 (7.5)	0 (0)		3 (8.3)	0 (0)	
	OAD	19 (39.6)	16 (40)	3 (37.5)		14 (38.9)	5 (41.7)	
	Insulin + OAD	23 (47.9)	19 (47.5)	4 (50)		18 (50)	5 (41.7)	
HT	Yes	22 (45.8)	17 (42.5)	5 (62.5)	0.442 ^F^	17 (47.2)	5 (41.7)	1.00 ^Y^
CRF	Yes	7 (14.6)	6 (15)	1 (12.5)	1.000 ^F^	6 (16.7)	1 (8.3)	0.662 ^F^
Hyperlipidemia	Yes	46 (95.8)	38 (95)	8 (100)	1.000 ^F^	34 (94.4)	12 (100)	1.000 ^F^

Results are expressed as mean ± standard deviation/median (minimum–maximum) or n (%). BMI: body mass index, DM: diabetes mellitus, OAD: oral antidiabetic, HT: hypertension, CRF: chronic renal failure. P^1^: Comparison of anatomically successful and unsuccessful groups. P^2^: Comparison of visually successful and unsuccessful groups, ^U^: Mann–Whitney U Test, ^Y^: Yates correction, ^t^: Independent Two Sample *t* test, ^P^: Pearson chi-square test, ^F^: Fisher’s Exact Test.

**Table 2:** Ocular parameters and their relationship with success.

		Total (n = 48)	Anatomical Success			Visual Success		
			Success (n = 40)	Unsuccess (n = 8)	P^1^	Success (n = 36)	Unsuccess (n = 12)	P^2^
Eye laterality	Right	23 (47.9)	19 (47.5)	4 (50.0)	1.000 ^Y^	15 (41.7)	8 (66.7)	0.243 ^Y^
	Left	25 (52.1)	21 (52.5)	4 (50.0)		21 (58.3)	4 (33.3)	
Lens	Phakic	39 (81.2)	32 (80)	7 (87.5)	1.000 ^Y^	28 (77.82)	11 (91.7)	0.416 ^F^
	Pseudophakic	9 (18.8)	8 (20)	1 (12.5)		8 (22.2)	1 (8.3)	
IOP (mmHg)	BT	15 (10–23)	15 (10–23)	15 (13–23)	0.460 ^U^	14.5 (10–21)	16 (12–23)	**0.013 ^U^**
	AT	17.5 (10–23)	17.5 (10–23)	18 (12–22)	0.425 ^t^	17 (10–23)	17 (10–22)	0.981 ^U^
P^3^		**0.040 ^Z^**	**0.008 ^Z^**	0.287 ^Z^		**<0.001 ^Z^**	0.406 ^Z^	
BCVA (LogMAR)	BT	0.44 (00–1.0)	0.48 (0.1–1.0)	0.25 (0–1.0)	0.236 ^U^	0.48 (0.1–1.3)	0.35 (0.1–1.0)	0.573 ^U^
	AT	0.18 (0.0–1.0)	0.18 (0–1.0)	0.19 (0–0.7)	0.856 ^U^	0.15 (0–0.78)	0.3 (0.1–1.0)	0.099 ^U^
P^4^		**˂0.001 ^Z^**	**<0.001 ^Z^**	0.109 ^Z^		**<0.001 ^Z^**	0.102 ^Z^	

Results are expressed as median (minimum–maximum) or n (%). BT: before treatment, AT: after treatment, BCVA: best-corrected visual acuity, IOP: intraocular pressure, P^1^: comparison of anatomically successful and unsuccessful groups. P^2^: Comparison of visually successful and unsuccessful groups; P^3^: comparison of intraocular pressure before and after treatment; P^4^: comparison of best corrected visual acuity before and after treatment. ^Y^: Yates correction; ^t^ independent two sample *t* test ^F^: Fisher’s exact test; ^U^: Mann–Whitney’s U Test; ^Z^: Wilcoxon test. *p* Value in bold type: statistically significant result.

**Table 3:** Quantitative optical coherence tomography parameters and their relationship with success.

			Anatomical Success			Functional Success		
			Success (n = 40)	Unsuccess (n = 8)	P^1^	Success (n = 36)	Unsuccess (n = 12)	P^2^
CMT (µm)	BT	427 (280–699)	435.5 ± 98.9	470.13 ± 103.2	0.374 ^t^	432 ± 89.9	469 ± 124.2	0.356 ^t^
	AT	312 (215–587)	314.5 ± 62.8	459.6 ± 105.4	**0.005** ^t^	303 (215–535)	420.5 (225–587)	**0.017 ^U^**
P^3^		**<0.001 ^Z^**	**<0.001 ^T^**	0.529 **^T^**		**<0.001 ^Z^**	**0.027 ^Z^**	
GCT (µm)	BT	31.5 (12–64)	33.3 ± 11.8	28.1 ± 12.8	0.272 ^t^	31.6 ± 10.4	34.9 ± 15.9	0.404 ^t^
	AT	25 (12–47)	25.1 ± 8.3	28.6 ± 11.5	0.311 ^t^	25.0 ± 7.3	27.7 ± 12.4	0.485 ^t^
P^3^		**<0.001 ^Z^**	**<0.001 ^T^**	0.849 **^T^**		**<0.001 ^T^**	0.072 ^T^	
IRT (µm)	BT	338.5 (195–620)	351.4 ± 99.1	371.8 ± 117.2	0.608 ^t^	344.7 ± 90.85	384.9 ± 127.5	0.328 ^t^
	AT	229.5 (90–486)	227.9 ± 63.1	376 ± 104.9	**0.005** ^t^	223.5 (90–448)	340 (172–486)	**0.022 ^U^**
P^3^		**<0.001 ^Z^**	**<0.001 ^T^**	0.760 **^T^**		**<0.001 ^Z^**	**0.015 ^Z^**	
ORT (µm)	BT	82 (75–122)	81.5 (75–122)	85.5 (75–105)	0.367 ^U^	81.5 (75–122)	82 (75–105)	0.867 ^U^
	AT	85 (70–107)	84 (70–107)	87 (78–101)	0.324 ^U^	83.6 ± 5.9	89.8 ± 8.8	**0.008 ^t^**
P^3^		0.287 ^Z^	0.271 ^Z^	0.799 ^Z^		0.806 ^Z^	**0.023 ^Z^**	
CCT (µm)	BT	265 (162–531)	264.5 (162–531)	296 (229–433)	0.193 ^U^	264 (162–497)	278 (168–531)	0.341 ^U^
	AT	251 (142–531)	246.5(142–531)	285 (238–430)	0.053 ^U^	250 (147–531)	264 (142–501)	0.398 ^U^
P^3^		0.560 ^Z^	**0.017 ^Z^**	0.484 ^Z^		0.270 ^Z^	0.071 ^Z^	

Results are expressed as mean ± standard deviation/median (minimum–maximum). BT: before treatment, AT: after treatment, CMT: central macular thickness, and GCT: ganglion cell thickness. IRT: inner retinal thickness, ORT: outer retinal thickness, CCT: central choroidal thickness, P^1^: comparison of anatomically successful and unsuccessful groups, P^2^: comparison of visually successful and unsuccessful groups, P^3^: comparison of pre- and post-treatment values. ^t^: independent two-sample *t* test, ^U^: Mann–Whitney U Test, ^Z^: Wilcoxon test and, ^T^: paired two-sample *t* test. *p* value in bold type: Statistically significant result.

**Table 4:** Qualitative optical coherence tomography parameters and their relationship with success.

		Total (n = 48)	Anatomical Success			Functional Success		
			Success (n = 40)	Unsuccess (n = 8)	P^1^	Success (n = 36)	Unsuccess (n = 12)	P^2^
Edema type	Cystoid	23(47.9)	18 (45)	5 (62.5)	0.454 ^F^	16 (44)	7 (58.3)	0.617 ^Y^
	Diffuse	25 (52.1)	22 (55)	3 (37.5)		20 (56)	5 (41.7)	
HE	Yes	26 (54.2)	23 (57.5)	3 (37.5)	0.442 ^F^	20 (55.6)	6 (50)	1.000 ^Y^
IRF	Yes	44 (91.7)	36 (90)	8 (100)	1.000 ^F^	33 (91.7)	11 (91.7)	1.000 ^F^
SRF	Yes	10 (20.8)	8 (20)	2 (25)	0.666 ^F^	7 (19.4)	3 (25)	0.695 ^F^
DRIL	Yes	12	10 (25)	2 (25)	1.000 ^F^	7 (19.4)	5 (41.7)	0.143 ^F^
EZ damage	Yes	18	14 (35)	4 (50)	0.451 ^F^	13 (36.1)	5 (41.2)	0.743 ^F^
HF	No	1 (2.1)	1 (2.5)	0 (0)	0.787 ^P^	1 (2.8)	0 (0)	0.915 ^P^
	D1	22 (45.8)	19 (47.5)	3 (37.5)		16 (44.4)	6 (50)	
	D2	18 (37.5)	15 (37.5)	3 (37.5)		14 (38.9)	4 (33.3)	
	D3	7 (14.6)	5 (12.5)	2 (25)		5 (13.9)	2 (16.7)	
Vitreomacular interface pathology	No	36 (75)	31 (77.5)	5 (62.5)	0.525 ^P^	27 (75)	9 (75)	0.777 ^P^
	ERM	3 (6.3)	2 (5)	1 (12.5)		3 (8.3)	0	
	PVD	3 (8.3)	3 (7.5)	1 (12.5)		3 (8.3)	1 (8.3)	
	VMA	2 (4.2)	1 (2.5)	1 (12.5)		1 (2.8)	1 (8.3)	
	VMT	3 (6.3)	3 (7.5)	0		2 (5.6)	1 (8.3)	

Results are expressed as n (%). HE: hard exudate, IRS: intraretinal fluid, SRF: subretinal fluid, DRIL: disorganization of the inner retinal layers, EZ: ellipsoid zone, HF: hyperreflective foci, ERM: epiretinal membrane, PVD: posterior vitreous detachment, VMA: vitreomacular adhesion, VMT: vitreomacular traction. P^1^: Comparison of anatomically successful and unsuccessful groups, P^2^: Comparison of visually successful and unsuccessful groups, ^F^: Fisher’s Exact test, ^P^: Pearson chi-square test, ^Y^: Yates correction.

**Table 5:** Peripheral blood parameters and their relationship with success.

		Anatomical Success			Visual Success		
		Success (n = 40)	Unsuccesss (n = 8)	P^1^	Success (n = 36)	Unsuccess (n = 12)	P^2^
Hb (g/dL)	13.5 ± 1.7	13.3 ± 1.8	14.2 ± 1.1	0.195 ^t^	13.45 ± 1.9	13.5 ± 1.2	0.893 ^t^
Hct (%)	40 ± 4.6	39.6 ± 4.7	42.2 ± 3.7	0.150 ^t^	39.9 ± 4.8	40.2 ± 4.0	0.852 ^t^
WBC (10^3^/µL)	8.19 (4.7–16.3)	8.27 (5.1–16.3)	6.69 (4.7–12.4)	**0.049 ^U^**	8.3 (4.7–14.7)	6.9 (5.4–16.3)	0.167 ^U^
Monocyte (1/µL)	580 (200–1320)	600 (300–1320)	530 (200–800)	0.542 ^U^	580 (200–1000)	565 (300–1320)	0.849 **^U^**
Monocyte/HDL	12.5 (3.9–25.8)	13.25 (5.6–22.8)	11.35 (3.9–25.8)	0.709 ^U^	14.0 (3.9–25.8)	11.2 (8–22.8)	0.821 **^U^**
BUN (mg/dL)	31.1 ± 12.3	31.9 ± 12.8	26.9 ± 9.0	0.294 ^t^	33.9 ± 12.08	22.6 ± 8.7	**0.005** ^t^
Creatinin (mg/dL)	0.87 (0.46–2.4)	0.9 (0.5–2.4)	0.88 (0.5–1.9)	0.740 ^U^	0.89 (0.46–2.4)	0.8 (0.5–1.1)	0.608 **^U^**
FBG (mg/dL)	162.5 (56–550)	159.5 (56–550)	216 (139–329)	0.218 ^U^	166 (62–550)	160 (56–331)	0.868 **^U^**
Triglyceride (mg/dL)	171 (45.7–593)	160 (45.7–593)	194.5 (99–319)	0.719 ^U^	148 (45.7–453)	209.5 (68–593)	0.353 **^U^**
HDL (mg/dL)	43 (28–98)	44.5 (28–98)	37 (31–51.6)	0.105 ^U^	41.5 (28–98)	45.5 (32–85)	0.712 **^U^**
LDL (mg/dL)	107 (14.3–300)	105 (14.3–300)	128.5 (90–183)	0.198 ^U^	106.3 ± 37.6	153.83 ± 61.05	**0.002** ^t^
HbA1c (%)	9.1 ± 2.2	9.2 ± 2.9	8.8 ± 2.9	0.660 ^t^	9.1 ± 2.15	9.2 ± 2.4	0.932 ^t^
ESR (mm/saat)	26.5 (4–90)	28.5 (4–90)	22.5 (15–41)	0.618 ^U^	24 (4–59)	30.5 (10–90)	0.404 **^U^**
CRP (mg/dL)	2.1 (0–14)	2.6 (0–14)	2 (0.7–7)	0.424 ^U^	2.5 (0–14)	2.1 (0.7–14)	0.847 **^U^**

Results are expressed as mean ± standard deviation or median (minimum–maximum). Hb: hemoglobin, Hct: hemotocrit, WBC: white blood cell, BUN: blood urea nitrogen, FBG: fasting blood glucose, HDL: high-density lipoprotein, LDL: low-density lipoprotein, HbA1c: glycosylated hemoglobin, ESR: erythrocyte sedimentation rate, CRP: C-reactive protein. P^1^: Comparison of anatomically successful and unsuccessful groups. P^2^: Comparison of visually successful and unsuccessful groups. ^t^: Two-sample *t* test. ^U^: Mann–Whitney U test. *p* value in bold type: statistically significant result.

**Table 6:** Results of binary logistic regression analysis for anatomical success.

		Anatomical Success			
		Success (n = 40)	Unsuccess (n = 8)	OR (%95 CI)	*p* Value
Age (year)		64.2 ± 8.85	63.13 ± 6.77	1.015 (0.928–1.11)	0.741
Gender	Female	17 (42.5)	5 (62.5)	0.443 (0.093–2.116)	0.308
	Male	23 (57.5)	3 (37.5)		
BMI (kg/m^2^)		28.8 ± 4.42	32.27 ± 5.04	0.83 (0.681–1.012)	0.065
DM duration (years)		16 ± 8.08	14.38 ± 6.97	1.027 (0.931–1.133)	0.591
HT	Yes	17 (42.5)	5 (62.5)	2.255 (0.473–10.759)	0.308
CRF	Yes	6 (15)	1 (12.5)	0.81 (0.084–7.819)	0.855
Lens	Phakic	32 (82.1)	7 (17.9)	0.571 (0.061–5.335)	0.623
	Pseudophakic	8 (88.9)	1 (11.1)		
BCVA (LogMAR)		0.53 ± 0.36	0.41 ± 0.42	2.907 (0.257–32.926)	0.389
IOP (mmHg)		15.35 ± 3.18	16.38 ± 3.62	0.91 (0.725–1.141)	0.414
Hb (g/dL)		13.3 ± 1.8	14.2 ± 1.1	0.736 (0.462–1.172)	0.196
Hct (%)		39.6 ± 4.7	42.15 ± 3.6	0.88 (0.738–1.049)	0.154
WBC (10^3^/µL)		8.4 ± 2.2	7.2 ± 2.4	1.437 (0.867–2.382)	0.160
Monosyt (1/µL)		591.25 ± 201.85	521.25 ± 215	1.002 (0.998–1.006)	0.371
Monosyt/HDL		13.3 ± 5.3	13.94 ± 7.0	0.979 (0.852–1.124)	0.762
BUN (mg/dL)		31.9 ± 12.8	26.9 ± 9.0	1.039 (0.968–1.115)	0.289
Creatinin (mg/dL)		0.89 ± 0.4	0.94 ± 0.41	0.764 (0.128–4.567)	0.768
FBG (mg/dL)		192.2 ± 103.5	219.9 ± 73.0	0.997 (0.99–1.004)	0.471
Triglycerid (mg/dL)		205.92 ± 132.49	199.25 ± 78.8	1 (0.994–1.007)	0.889
HDL (mg/dL)		47.7 ± 14.3	39.65 ± 8.1	1.074 (0.979–1.178)	0.133
LDL (mg/dL)		115.5 ± 50.6	131.5 ± 36.6	0.994 (0.979–1.008)	0.398
HbA1c (%)		9.2 ± 2.1	8.8 ± 2.85	1.089 (0.752–1.575)	0.652
ESR (mm/saat)		30 ± 18.1	25.25 ± 9.2	1.019 (0.968–1.073)	0.467
CRP (mg/dL)		4.0 ± 3.7	2.7 ± 2.0	1.159 (0.853–1.574)	0.346
Edema type	Cystoid	18 (78.3)	5 (21.7)	0.491 (0.103–2.339)	0.372
	Diffuse	22 (88)	3 (12)		
CMT (µm)		435.5 ± 98.99	470.13 ± 103.16	0.997 (0.989–1.004)	0.369
GCT (µm)		33.25 ± 11.75	28.13 ± 12.77	1.041 (0.969–1.119)	0.270
IRT (µm)		351.35 ± 99.13	371.75 ± 117.21	0.998 (0.991–1.005)	0.601
ORT (µm)		83.93 ± 8.44	86.63 ± 9.9	0.969 (0.897–1.047)	0.427
CCT (µm)		272.88 ± 81.56	308.25 ± 79.02	0.995 (0.987–1.004)	0.269
HE (Yes)		23 (88.5)	3 (11.5)	0.443 (0.093–2.116)	0.308
SRF (Yes)		8 (80.0)	2 (20.0)	1.333 (0.225–7.89)	0.751
DRIL (Yes)		10 (83.3)	2 (16.7)	1.333 (0.225–7.89)	0.751
EZ Damage (Yes)		14 (77.8)	4 (22.2)	1.857 (0.402–8.582)	0.428

Results are expressed as mean ± standard deviation/median (minimum–maximum) or n (%). BMI: body mass index, DM: diabetes mellitus, HT: hypertension, CRF: chronic renal failure, BCVA: best corrected visual acuity, IOP: intraocular pressure, Hb: hemoglobin, Hct: hemotocrit, WBC: white blood cell, BUN: blood urea nitrogen, FBG: fasting blood glucose, HDL: high-density lipoprotein, LDL: low-density lipoprotein, HbA1c: glycosylated hemoglobin, ESR: erythrocyte sedimentation rate, CRP: C-reactive protein, CMT: central macular thickness, GCT: ganglion cell thickness, IRT: inner retinal thickness, ORT: outer retinal thickness, CCT: central choroidal thickness, HE: hard exudate, SRF: subretinal fluid, DRIL: disorganation of the inner retinal layers, and EZ damage: photoreceptor inner segment–outer segment.

**Table 7:** Binary logistic regression analysis results for visual success.

		Functional Success			
		Success (n = 36)	Unsuccess (n = 12)	OR (%95 CI)	*p*
Age (years)		63.89 ± 8.95	64.42 ± 7.23	0.992 (0.918–1.073)	0.850
Gender	Female	15 (41.7)	7 (58.3)	0.51 (0.136–1.92)	0.320
	Male	21 (58.3)	5 (41.7)		
BMI (kg/m^2^)		28.9 ± 4.8	30.9 ± 4.1	0.906 (0.779–1.053)	0.198
DM duration (years)		16.6 ± 7.95	13.1 ± 7.25	1.062 (0.972–1.16)	0.182
HT	Yes	17 (47.2)	5 (41.7)	0.798 (0.213–2.992)	0.738
CRF	Yes	6 (16.7)	1 (8.3)	0.455 (0.049–4.214)	0.488
Lens	Phakic	28 (77.8)	11 (91.7)	0.318 (0.036–2.851)	0.306
	Pseudophakic	8 (22.2)	1 (8.3)		
BCVA (LogMAR)		0.53 ± 0.38	0.46 ± 0.37	1.67 (0.256–10.891)	0.592
IOP (mmHg)		14.81 ± 2.77	17.67 ± 3.73	0.756 (0.606–0.943)	**0.013**
Hb (g/dL)		13.45 ± 1.86	13.53 ± 1.19	0.973 (0.662–1.431)	0.890
Hct (%)		39.93 ± 4.83	40.22 ± 4.04	0.986 (0.854–1.138)	0.848
WBC (10^3^/µL)		8.22 ± 1.92	7.98 ± 3.08	1.052 (0.771–1.435)	0.751
Monosyt (1/µL)		573.06 ± 183.6	599.17 ± 262.73	0.999 (0.996–1.003)	0.698
Monosyt/HDL		13.59 ± 5.7	12.87 ± 5.07	1.025 (0.908–1.157)	0.691
BUN (mg/dL)		33.9 ± 12.08	22.59 ± 8.67	1.113 (1.028–1.206)	**0.008**
Creatinin (mg/dL)		0.94 ± 0.45	0.8 ± 0.16	3.068 (0.325–28.95)	0.328
FBG (mg/dL)		199.17 ± 103.53	189.67 ± 87.44	1.001 (0.994–1.008)	0.771
Triglyceride (mg/dL)		191.58 ± 111.67	244.5 ± 155.68	0.997 (0.992–1.002)	0.208
HDL (mg/dL)		45.72 ± 13.29	48.13 ± 15.57	0.988 (0.944–1.034)	0.597
LDL (mg/dL)		106.28 ± 37.57	153.83 ± 61.05	0.977 (0.96–0.995)	**0.013**
HbA1c (%)		9.11 ± 2.15	9.18 ± 2.37	0.987 (0.73–1.333)	0.930
ESR (mm/saat)		27.56 ± 15.14	34.17 ± 21.5	0.978 (0.941–1.016)	0.250
CRP (mg/dL)		3.82 ± 3.5	3.74 ± 3.61	1.006 (0.832–1.218)	0.947
Edema type	Cystoid	16 (69.6)	7 (30.4)	0.571 (0.152–2.145)	0.407
	Diffuse	20 (80)	5 (20)		
CMT (µm)		432.03 ± 89.86	469 ± 124.21	0.996 (0.99–1.003)	0.266
GCT (µm)		31.56 ± 10.42	34.92 ± 15.95	0.977 (0.924–1.032)	0.397
IRT (µm)		344.69 ± 90.85	384.92 ± 127.46	0.996 (0.99–1.003)	0.236
ORT (µm)		84.64 ± 9.01	83.58 ± 7.77	1.016 (0.935–1.104)	0.713
CCT (µm)		271.67 ± 75.4	300.08 ± 97.81	0.996 (0.988–1.004)	0.299
HE (Yes)		20 (76.9)	6 (23.1)	0.800 (0.216–2.961)	0.738

Results are expressed as mean ± standard deviation/median (minimum–maximum) or n (%). BMI: body mass index, DM: diabetes mellitus, HT: hypertension, CRF: chronic renal failure, BCVA: best corrected visual acuity, IOP: intraocular pressure, Hb: hemoglobin, Hct: hemotocrit, WBC: white blood cell, BUN: blood urea nitrogen, FBG: fasting blood glucose, HDL: high-density lipoprotein, LDL: low-density lipoprotein, HbA1c: glycosylated hemoglobin, ESR: erythrocyte sedimentation rate, CRP: C-reactive protein, CMT: central macular thickness, GCT: ganglion cell thickness, IRT: inner retinal thickness, ORT: outer retinal thickness, CCT: central choroidal thickness, HE: hard exudate. *p* value in bold type: statistically significant result.

## Data Availability

The data supporting this study’s findings are available from the corresponding author upon request.

## Abbreviations

BCVA—Best Corrected Visual Acuity, BUN—Blood Urea Nitrogen, CMT—Central Macular Thickness, CCT—Central Choroidal Thickness, DME—Diabetic Macular Edema, DM—Diabetes Mellitus, DR—Diabetic Retinopathy, GCT—Ganglion Cell Layer Thickness, HbA1c—Glycosylated Hemoglobin, HDL—High-Density Lipoprotein, IOP—Intraocular Pressure, IRT—Inner Retinal Thickness, IVB—Intravitreal Bevacizumab, LDL—Low-Density Lipoprotein, NPDR—Non-Proliferative Diabetic Retinopathy, OCT—Optical Coherence Tomography, ORT—Outer Retinal Thickness, VEGF—Vascular Endothelial Growth Factor, WBC—White Blood Cell.
